# Intensive Adoption as a Management Strategy for Unowned, Urban Cats: A Case Study of 25 Years of Trap–Assess–Resolve (TAR) in Auckland, New Zealand

**DOI:** 10.3390/ani12172301

**Published:** 2022-09-05

**Authors:** Michael C. Calver, Heather M. Crawford, Fiona R. Scarff, J. Stuart Bradley, Peter Dormon, Samantha Boston, Patricia A. Fleming

**Affiliations:** 1 Environmental and Conservation Sciences, Murdoch University, 90 South Street, Murdoch, WA 6150, Australia; 2 Lonely Miaow Association, Lonely Miaow Association, 35a, Riddell Road, Glendowie, Auckland 1071, New Zealand; 3 Harry Butler Institute, Murdoch University, 90 South Street, Murdoch, WA 6150, Australia

**Keywords:** TNR, free-roaming cat, stray cat, euthanasia, cat welfare, adoption

## Abstract

**Simple Summary:**

Unowned urban cats may suffer from poor welfare and cause problems, e.g., public health risks, nuisances, and urban wildlife predation. For 25 years, the Lonely Miaow (Inc.) charity in Auckland, New Zealand, has used intensive adoption to rehome unowned cats. By the end of 2019, LM volunteers had trapped 14,611 unowned cats, which were adopted wherever possible (64.2%), euthanized if unsocialised or in grave ill-health (22.2%), or (infrequently) neutered and returned to the site (5.7%). The remaining 7.9% had other outcomes, such as being transferred to other shelters. Adoption rates increased over time, exceeding 80.0% in 2018 and 2019. The cost of processing each cat from capture to adoption rose from NZD 58 in 1999 to NZD 234 in 2017. Approximately 80% of colonies (sites where cats were trapped) were around residential areas. Most cats were young and very few were over 5 years old. Around one in five cats needed veterinary treatment, with respiratory infections and injury common. Adopting cats and removing them from the streets improves their welfare, essentially benefitting the community and the cats. The effectiveness of adoption strategies would be enhanced by fewer abandonments of owned cats and kittens, fitting within integrated strategies for the control of unowned cats involving community education.

**Abstract:**

Globally, unowned urban cats are a major concern because they may suffer from poor welfare and cause problems, including public health risks, nuisances, and urban wildlife predation. While management options are often presented as a choice between culling or trap–neuter–return (TNR), for 25 years, the Lonely Miaow (Inc.) charity in Auckland, New Zealand (hereafter LM), has used a third strategy—intensive adoption or trap–assess–resolve (TAR). As of 2019, of 14,611 unowned cats trapped, 64.2% were adopted, 22.2% were euthanized if unsocialised or in grave ill-health, 5.7% were neutered and returned to the site, and 7.9% had other outcomes, such as being transferred to other shelters. Adoption rates increased over this time, exceeding 80.0% in 2018 and 2019. The cost of processing each cat from capture to adoption rose from NZD 58 in 1999 to NZD 234 by 2017. Approximately 80% of colonies (sites where cats were trapped) were around residential areas. Approximately 22% of cats required veterinary treatment after capture; common ailments included respiratory infections, ringworm, dental problems, and trauma. Consistently, 52% of cats were young kittens (<10 weeks old), *c*. 80% of cats were <1 year old, and only *c*. 2% were estimated to be >5 years old. TAR avoids euthanasia where possible. Its effectiveness would be enhanced by fewer abandonments of owned cats and kittens, fitting within integrated strategies for the control of unowned cats involving community education. Cat adoptions improve the welfare of cats and, with appropriate husbandry, should alleviate concerns about nuisances, public health, and attacks on wildlife or the cats themselves, essentially benefitting the community and the cats. This case study is relevant to other cities around the world that are seeking to manage unowned cats.

## 1. Introduction

**Figure 1:**
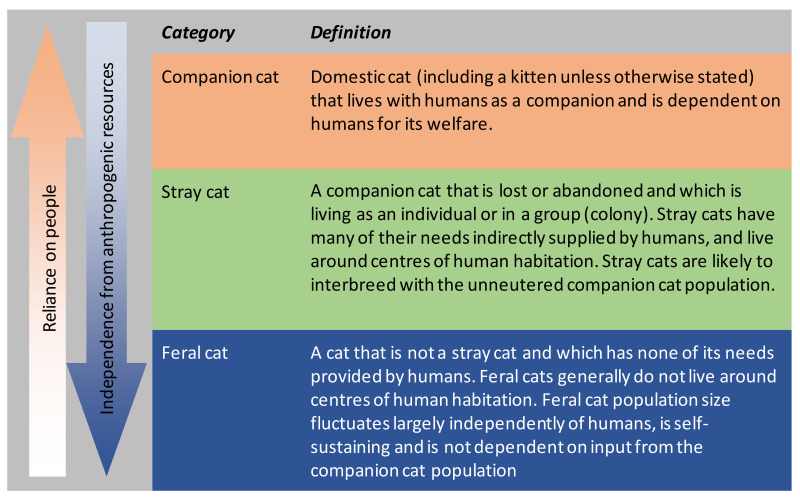
Categories of cats under definitions provided by the New Zealand Government’s *Animal Welfare (Companion Cats) Code of Welfare* 2018. Sexually entire cats may interbreed across categories.

Domestic cats, *Felis catus*, are prolific breeders, producing litters of up to four kittens two to three times a year [1], (Chapter 4). Thus, one unfortunate consequence of their global popularity as companion animals [2,3] is the potential for large populations of unowned, free-ranging cats around human habitations, sustained by the abandonment of owned animals and uncontrolled breeding [4,5,6,7,8]. They are variously named unowned cats, free-ranging cats, stray cats, community cats, or feral cats, with little agreement on a chaotic nomenclature [9,10]; see Figure 1 for the nomenclature that we use herein. The numbers of unowned cats are difficult to quantify, with estimates of their abundance ranging between 30 and 100 million in the USA (Ref. [11] and included references), 700,000 in Australia [12], and 196,000 in New Zealand [13]. Depending on the geographic region, stray cats may suffer from similar poor welfare outcomes to free-ranging owned cats, including road accident trauma [14,15,16,17], poisoning [18], ingestion of hazardous garbage [7], predation by larger carnivores [19], disease [20], and human persecution [21]. Free-ranging cats may also kill or harass wildlife [22,23,24], including threatened species [25,26,27], present a disease risk to people, pets, and wildlife [28,29,30,31,32], and annoy people [1,33]. Therefore, the management of stray cats is an ethical and community concern.

While the scale of the problem of stray cat management requires intervention, there is an extensive debate regarding the most effective and ethical options (e.g., contrast [34] with [1], or [35,36,37] with [8,38]). Options are often constrained by legislation, which varies greatly between jurisdictions [39,40,41,42,43]. Trapping and euthanizing cats (TE) [37,44] sometimes meets with community resistance. There has been a growing number of literature reports describing alternative actions for potentially resolving stray cat issues, especially regarding trapping, neutering, and returning cats to the site of capture, e.g., trap–neuter–return (TNR); with variants such as TNVR (where cats are vaccinated before release) or TTNR (where there is intense targeting of a restricted geographical area), e.g., [45,46]. These methods entail varying degrees of follow-up support, including substantial time and money invested by ‘caretakers’ for these cats. There are strong advocates and opponents of TNR in all its options [8,35,36,37,38].

New Zealand has very high cat ownership rates, with *c*. 35% of households owning one or more cats [47]. In common with many other countries, the popularity of cats as pets has also contributed to large populations of unowned cats [43,48]. In response to concerns about their welfare and the problems they may cause (e.g., [48,49]), since 1990 the Lonely Miaow Association, Inc., Stray Cats New Zealand Trust (hereafter LM; https://www.lonelymiaow.co.nz (accessed on 1 August 2022)) based in Auckland (New Zealand’s largest city), has operated a trap–assess–resolve (TAR) approach (also called ‘capture and rehoming’, *sensu* [50]) to manage unowned, free-ranging cats. Volunteers trap colonies of unowned cats, which are adopted wherever possible or euthanized if they are unsocialised or in grave ill-health. The aim is to control the population with low euthanasia without returning cats to life on the streets.

In this paper, we present records from the history of LM’s operations to address:

- Where unowned cat colonies are located.
- Age structures of the trapped cats.
- Outcomes for cats, including analysis by age (kitten or adult).
- Health assessment of cats.
- Prevalence of neutering amongst unowned cats as a conservative indicator of loss or abandonment.
- The costs incurred by LM and for what purposes.

We then discuss the advantages and disadvantages of TAR relative to two other widely-used management options for stray cats: TE and TNR. The data and discussion form a case study in implementing TAR relevant to urban areas globally where populations of unowned cats are managed.

## 2. Materials and Methods

### 2.1. Context: Managing Unowned Cats in New Zealand

The broad context of animal management in New Zealand, including owned and unowned cats, is outlined in the New Zealand Animal Welfare Strategy [51], (p. 4). The primary legislation enforcing the strategy is the *Animal Welfare Act 1999* [52], including provisions for codes of welfare that, where applied to individual species, state the minimum standards for care and recommendations for best practices. Sumner and colleagues [43] note that New Zealand legislation is clearer with regard to New Zealand’s feral cats than it is for stray cats (in New Zealand, as in Australia, feral cats are those remote from human habitation and with no human interaction [10]), so they argue for national legislation for managing all categories of cats.

### 2.2. History of Lonely Miaow Association Inc.

LM is a non-profit group that was founded by Peter Dormon in 1990 and incorporated in 1995. With a general goal of ‘no more strays’, LM runs three main activities across the Auckland metropolitan area: provision of cat care information, rehoming strays, and assisting landholders seeking the removal of stray cats.

The rehoming and assistance activities carried out by LM involve TAR in line with the provisions of New Zealand’s animal welfare code and strategy [51,52]. Following a request from the public to remove stray cats, volunteers undertake trapping. If necessary, they arrange veterinary treatment for trapped cats, including euthanasia for any that are seriously ill, injured, or too unsocialised for adoption, as judged by both the person who traps the cats and the veterinarian who examines them (see Supplementary Material Table S1 for schedules for assessment, plus an example media interview featuring LM volunteers). Other cats are then placed in foster homes, where ongoing care may include socialisation or administering medication/wound dressing under veterinary direction. All foster carers and their premises are assessed via a home visit and induction before joining the program. Before being advertised online for adoption, all cats are neutered, treated for worms and fleas, and given at least their initial vaccinations for panleukopenia and two strains of cat influenza. Since 2010, following the advent of the New Zealand Companion Animal Register in 2007, LM began microchipping and registering cats. Today, all cats are microchipped before adoption and registered in the name of the new owner.

### 2.3. Collation of Lonely Miaow and Veterinary Records

Summary data tables were extracted by Peter Dormon and Samantha Boston from the LM database. No data were available for 1990–1995 and only limited data were available for the period 1995–1998. Assessments focused on:

1. The site locations where stray cats were trapped (colonies in the LM records) following requests to remove them by landholders—these were classified as being situated in: residential properties, commercial premises, countryside/farms, industrial sites, other (various small categories, including restaurants, hospitals, seaside, council-subsidised housing, and schools). Data for locations were provided for the date range July 1995 to June 2020.

Other records were provided between July 1999 and December 2019, detailing:

2. Estimated age—five categories: young kitten (<10 weeks old), older kitten (10 weeks to 6 months), young adult (6 months to 1 year), adult (1–5 years), senior cat (>5 years old).
3. Outcome—adopted (including cats adopted by private individuals, adopted by their fosterers, or taken by pet shops), euthanized, returned to the site, other (including cats found dead, transferred to other shelters, died during operations or from sickness, or escaped).
4. Costs—veterinary, food, litter, microchipping, other.

LM uses multiple veterinary clinics across Auckland, with the choice of clinic often a matter of convenience for LM volunteers. The owner of one of these clinics, the Kohimarama Veterinary Clinic, kindly made available the clinical records for LM cats treated in the clinic in 2000 and between January 2008 and April 2018. We extracted and tabulated data from these clinical records. All cats not recommended for euthanasia were routinely wormed, treated for fleas, neutered if they were entire, and given their first vaccinations for panleukopenia and two strains of cat influenza, so we recorded the prevalence of other conditions. Data on the health of cats were noted on the date of first presentation, so that if a cat developed a condition while in foster care that condition was not recorded. Histories over time could be identified from case numbers or from notes indicating that the cat had been presented before. We also noted if females were pregnant, lactating, or had engorged mammaries, as well as any evidence that a cat had been owned previously (e.g., already neutered, microchipped). Cats were not routinely checked for feline immunodeficiency virus (FIV) or feline leukaemia virus (FeLV), although when tests were requested, we recorded the results.

LM also provided receipts for services conducted by a further 26 veterinary clinics across Auckland between 2010 and 2014 that treated cats at first presentation and during foster care. The receipts were examined for indications of likely conditions treated by these clinics, as revealed by the medications used or treatments described.

### 2.4. Statistical Analyses

Most questions from LM data or veterinary clinical histories were answered with a crosstabulation of data, including chi-square tests where appropriate. The effects of the age of the cats (the five age categories), their sex (male or female), and the year in which they were processed (1999–2019, ignoring the low numbers of cats handled in earlier years) on the likelihood of adoption were assessed using generalised linear models (GLM) in SPSS 22 for Windows [53]. The probability of adoption was initially modelled using a binomial distribution and probit link function, including predictor factors: cat age, cat sex, year, and age x sex interaction. To further investigate the interaction, a second probit analysis used an age variable with two levels (kitten and adult) and omitted the sex of the cats. To explore how the costs of TAR changed with the scale of the operation, Pearson’s correlation coefficients were calculated relating unit costs to the number of cats processed annually, with two-tailed tests for differences from zero.

## 3. Results

### 3.1. Colonies Resolved, Age Profiles, and Outcomes for Trapped Cats 1995–2020

Between July 1995 and June 2020, 3737 colonies (each defined as a specific callout from a landholder, usually in response to cats aggregating at a place providing shelter or food) were processed using TAR. The data cannot determine whether all these colonies were spatially or temporally independent, so some migration might have been possible between some of them. The data also include multiple callouts to the same location. Most were on residential properties (79.1% in 1995–2010, 81.2% in 2011–2020) (Table 1). There was a slight change in the relative proportions of colonies in the different categories over time, because of a small drop in the proportion of cats caught at commercial premises or in the peri-urban environment, and a small increase in the proportion of cats trapped at industrial sites or council-subsidised housing in 2011–2020 (χ^2^_6_ = 54.9, *p* < 0.001).

In total, 14,611 cats were processed through LM between July 1995 and December 2019. The mean number of cats processed per annum was 695 ± 332 s.d. (calculated from 1999 when annualised records began). The overall sex ratio was 45.6% males and 54.4% females for the 13,265 cases where sex was recorded. The sex ratio did not change significantly over time (χ^2^_21_ = 16.10, *p* = 0.762). However, the sex ratio did change with the age of the animal. There was an equal sex ratio for young kittens (50.0% male). This shifted to a bias towards females for young adults (32.5% male) and adults (37.8% male), before returning to a bias to males for seniors (58.9% male) (χ^2^_4_ = 227.16, *p* < 0.001).

Age data were available for most (98.8%) cats. Overall, *c*. two-thirds were kittens: 51.9% as young kittens (<10 weeks) or 16.1% as older kittens (10 weeks–6 months). The remaining third were adults: 9.2% young adults (6 months–1 year), 19.7% adults (1 year–5 years), and 1.9% seniors (>5 years).

**Figure 2:**
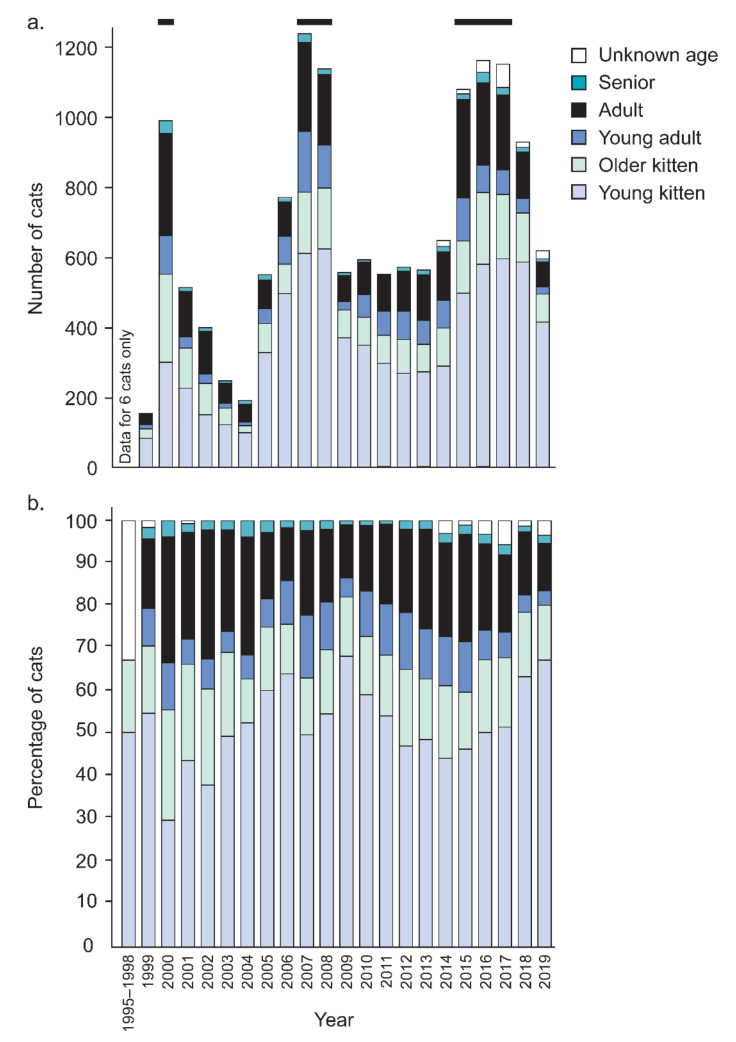
The age of cats processed for trap–assess–resolve (TAR) through Lonely Miaow, Auckland, New Zealand, between July 1995 and December 2019, showing the breakdown by year. Rectangles at the top highlight 6 years where efforts exceeded ~1000 cats per annum. The exact numbers are shown in (**a**) and the percentages in (**b**).

There were 6 years when the number of cats processed annually reached or exceeded ~1000: 2000, 2007, 2008, and 2015–2017 (Figure 2a). During these years, there were marginally more adult cats (34.1%) processed through the system than the overall value (30.8%), while kittens averaged 64.2% compared to the overall value of 68.0% of all cats processed (Figure 2b).

**Figure 3:**
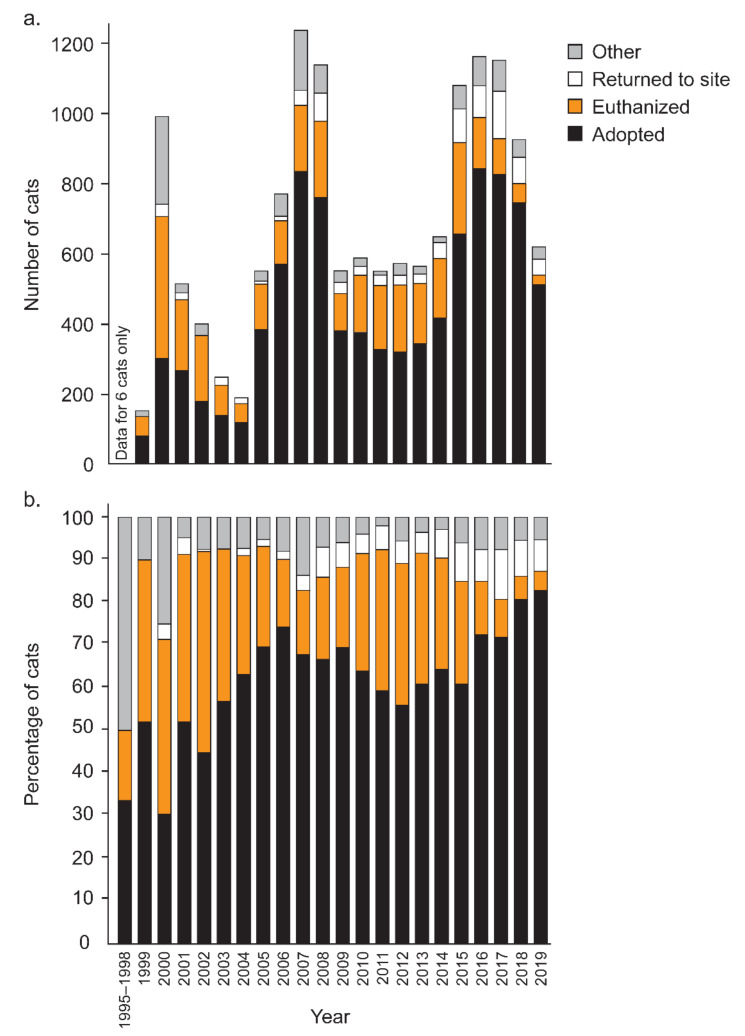
The fate of cats processed for trap–assess–resolve (TAR) through Lonely Miaow, Auckland, New Zealand, between July 1995 and December 2019, showing the breakdown by year. ‘Adopted’ includes adoptions brokered by Lonely Miaow, cats adopted by a foster carer, or cats placed with a pet shop. ‘Other’ includes cats that died, were found dead, escaped, or were transferred to another shelter, and ‘unknown’ where a volunteer had not entered the data. The exact numbers are shown in (**a**) and the percentages in (**b**).

The fate of cats processed through LM shifted markedly over time, with the proportion of cats being adopted nearly doubling from 43.4% of cats in the first decade of establishment to 72.5% over the last five years (Figure 3). During this time, the proportion of cats euthanized decreased from 40.0% in the first decade of establishment to only 12.1% over the last five years.

**Figure 4:**
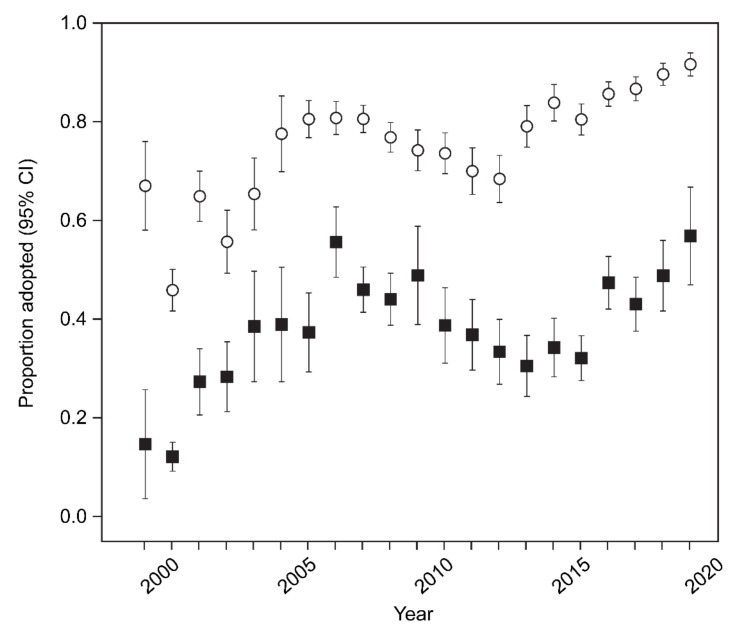
The proportion of kittens and adult cats adopted between 1999 and 2019 through the trap–assess–resolve (TAR) program run by Lonely Miaow, Auckland, New Zealand. Open symbols represent kittens and filled symbols represent adults. Error bars represent 95% confidence intervals.

Younger cats were more likely to be adopted than older cats (probit GLM model including cat age, cat sex, year, and age x sex interaction: Wald chi-square df 4 = 2285.2, *p* < 0.001), and annual adoptions increased over time (Wald chi-square df 1 = 179.3, *p* < 0.001). Overall, females were more likely to be adopted (Wald chi-square df 1 = 6.9, *p* = 0.009). The adoption rates of female cats versus male cats changed with age, with male kittens more likely to be adopted than female kittens and adult females more likely to be adopted than adult males (Wald chi-square df 4 = 30.0, *p* < 0.001). To further investigate the interaction, a second probit analysis used an age variable with two levels (kitten and adult) and treated time as a categorical variable with four periods each of five years. We omitted the sex of the cats. It confirmed strongly that kittens were more likely to be adopted than adults (Wald chi-square df 1 = 1758.9, *p* < 0.001), annual adoptions changed with the period (Wald chi-square df 3 = 525.2, *p* < 0.001), and the adoption rate of kittens increased more rapidly with time than adults (Wald chi-square df 3 = 50.3, *p* < 0.001) Figure 4. Examining kittens and adults separately, the effect of sex was significant in kittens, where 82.0% of females were adopted as opposed to 84.1% of males (Wald’s chi-square = 8.5, df 1, *p* = 0.003). In adults, sex was also significant (Wald’s chi-square = 13.2, df = 1, *p* = 0.001), with 42.5% of females adopted, as opposed to 36.7% of males, a reversal of the previous bias.

### 3.2. Health Assessments

**Figure 5:**
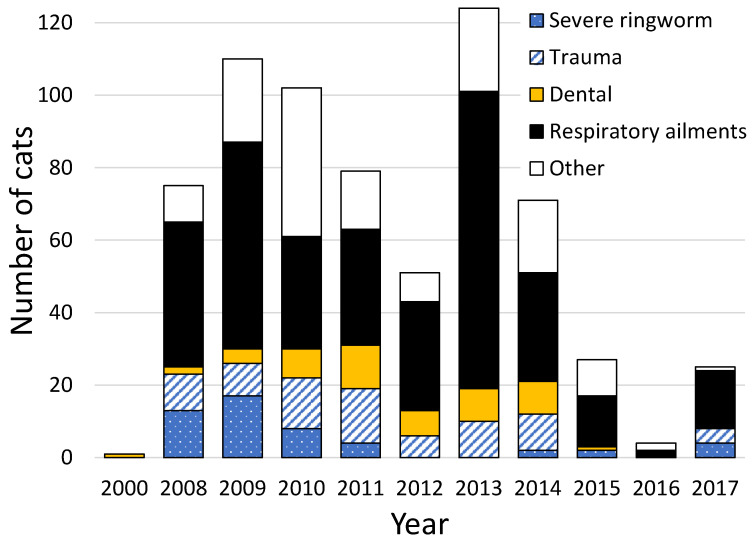
Ailments for which 541 of 2470 cats processed for trap–assess–resolve (TAR) were treated by the Kohimarama Veterinary Clinic in 2000 and between 2008 and 2018 as part of the processing for Lonely Miaow, Auckland, New Zealand. Note that a cat could be treated for multiple conditions, so the sum of the bars in the figure exceeds 541.

Excluding microchipping, neutering, flea and worm treatments, and vaccinations, 541 (21.9%) of LM cats who presented to the Kohimarama Veterinary Clinic in 2000 and 2008–2020 required veterinary treatment on the first visit (Figure 5). Common ailments included symptoms of respiratory tract infections indicative of cat influenza (eye or nasal discharges, rattly chest, sneezes) (61.7%), trauma including fractures, amputations, gloving injuries (where the skin is stripped from a limb or the tail) and fight wounds (14.4%), dental problems, including gingivitis, broken teeth, and periodontitis (9.8%), and ringworm (9.2%). Overall, 63 cats were tested for FIV (41.3% tested positive) and 17 for FeLV (23.5% tested positive). Given that not every cat was tested, these results do not indicate the prevalence of FIV and FeLV in the Auckland stray cat population. A total of 80 (15.0%) of the 452 female cats were neutered although pregnant, aborting the kittens. A further 37 females (8.2%) had recently given birth because they had engorged mammaries indicative of recent feeding of kittens. Every effort was made to trap kittens with their mothers.

Receipts from a further 26 veterinary practices between 2010 and 2014 showed that the main conditions for cats at first presentation or in foster care were:

- Respiratory and eye infections indicative of cat influenza.
- Skin infections, mainly ringworm.
- Urinary tract infections.
- Severe worm infestations requiring repeated and intensive worming, often associated with diarrhoea and dehydration.
- Secondary bacterial infections associated with cat influenza or diarrhoea.

There was evidence of significant discounting of fees on most occasions.

### 3.3. Estimation of Abandonment Rates

A total of 31 cats (1.2% of initial presentations to the Kohimarama Veterinary Clinic in 2000 and 2008–2018) were identified as having been owned previously, because when they were trapped they were already neutered and were not ear-tipped or tattooed to identify them as part of a TNR program (26 cats), had a microchip (2 cats), had dental work that had evidently been done by a veterinarian (1 cat), was judged so friendly and socialised that it must have been owned recently (1 cat), or the owner was traced (method not clear, 1 cat). Given that these cats must have been lost or abandoned, they gave a minimum baseline indication of loss or abandonment in the Auckland stray cat population. While this figure is low, the real rate will be increased by the loss or abandonment of entire animals that are not microchipped, which could not be detected.

### 3.4. Costs

**Figure 6:**
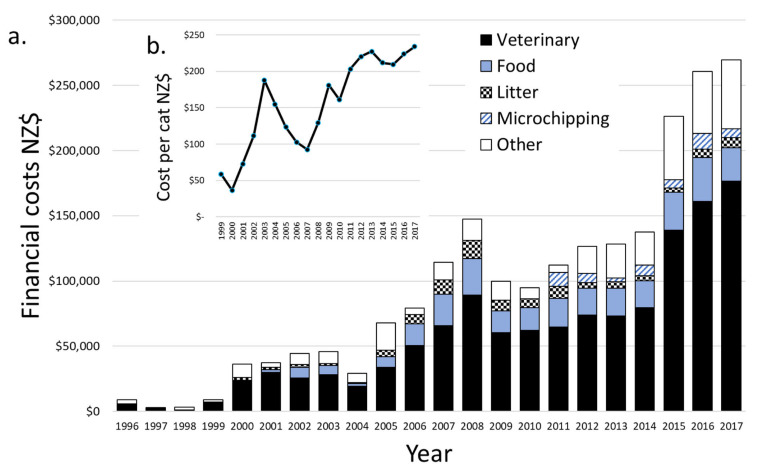
The financial costs of processing 13,062 stray cats (where reliable data were available) processed for trap–assess–resolve (TAR) through Lonely Miaow, Auckland, New Zealand, between 1996 and 2017, (**a**) by category of cost and (**b**) average estimated per cat.

Between 1996 and 2017, NZD 2.1 million was spent supporting the TAR program. Annual expenditure grew from NZD 8879 in 1996 to NZD 269,451 in 2017. Most costs (61.1%) were veterinary (Figure 6). However, we note that the contributions of unpaid volunteers, donations of consumables, such as cat litter, and the pro bono or discounted work of some veterinarians are not included in these figures. Estimates of volunteer hours and pro bono veterinary contributions could be assessed by asking participants to keep diaries of hours, which could then be costed at an agreed rate.

**Figure 7:**
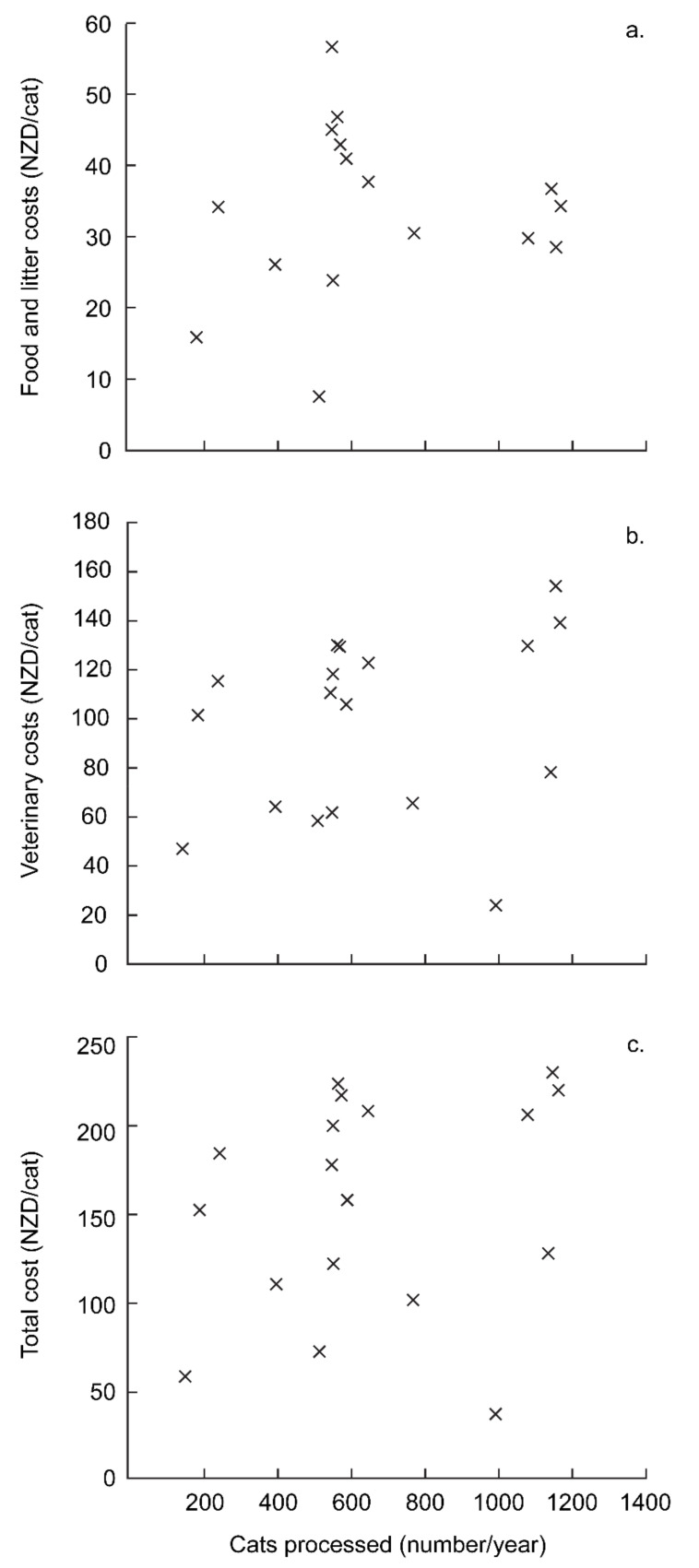
Per capita costs in relation to the number of cats processed for trap–assess–resolve (TAR) through Lonely Miaow, Auckland, New Zealand. Each point represents a year, from 1999–2017. (**a**) gives food and litter costs, (**b**) gives veterinary costs and (**c**) gives total costs. All costs are in NZD.

From 1999 (when annualised case records began) to 2017, the average costs per cat rose from NZD 58 to NZD 234 (Figure 6 inset). Food and litter costs per cat did not change with the annual number of cats processed (Figure 7a, r = 0.049, *p* = 0.853). Veterinary costs per cat increased strongly (Figure 6a), suggesting that more thorough care was being provided, the prevalence or severity of ailments rose, veterinary costs rose at around triple the general rate of inflation (RBNZ 2021), or that there was reduced fee discounting or pro bono work. Total and veterinary costs per cat were not related to the number of cats processed each year (Figure 7b,c total: r = 0.113, *p* = 0.646 vet: r = 0.109, *p* = 0.657). This indicates that as LM grew, there were, in net, no substantial economies of scale; either positive (e.g., bulk discounts) or negative (e.g., saturating the capacity of pro bono services).

## 4. Discussion

The data arising from the LM program are relevant in terms of assessing the welfare of stray cats in Auckland, as well as evaluating the advantages and disadvantages of TAR relative to TNR and TE in terms of costs and the range of problems related to stray cats that they solve. These data represent detailed insights into the lives of stray cats on the streets of a developed country recognised for its high value in animal welfare [51,52]. The study also captures the enormous efforts of veterinary professionals, volunteers, providers of pro bono services, and an enduring not-for-profit organisation in neutering, medicating, and rehoming stray cats.

### 4.1. Distribution of Stray Cats in Auckland

LM responds to requests from the public to remove stray cats, so the distribution of colonies likely reflects, to an unknown extent, the distribution of complaints to areas of a higher population rather than the distribution of cats. Most LM colonies over the period 1995–2020 came from residential areas (81% for 2011–2020 and 79% for 1995–2010 in the present study; 75% for 1991–2011 [54]. Thus, there is either a concentration of colonies in residential areas over time or increased reports of colonies there relative to other areas. The increase in the number of colonies reported over time could reflect increased awareness of LM services, increased public awareness of stray cats and concern for them, or possibly increases in stray cat numbers.

Separate cat collection records in Auckland revealed that strays were geographically clustered, with possibly a higher prevalence in economically-deprived areas [54]. In that study, colonies were associated with residential areas of higher human population density and, more weakly, higher levels of social deprivation that could be targeted with education campaigns regarding the management of stray cats, as well as subsidies to assist in trapping, neutering, and rehoming. Particular attention could be given to any problems caused by provisioning these cats. The idea of identifying communities where education would be effective could be applied internationally, with a growing body of education approaches addressing barriers to good husbandry (e.g., [55,56,57,58,59]).

### 4.2. Welfare of Stray Cats in Auckland

The welfare of stray cats is an important concern in deciding whether and how to manage their populations. Visual assessments of cats conducted at varying distances are commonly used to note such factors as body conditions, coat conditions, injuries, and prominent eye or nasal discharges (e.g., [6,60]). However, they are less commonly calibrated against actual veterinary examinations as proposed by [48]. Where detailed records are kept of the health of cats admitted to shelters or processed in TNR programs [61,62], or cadavers are examined following euthanasia (e.g., [7]), data indicate compromised animal welfare. Therefore, although some authors concluded that the managed stray cats were in good health (e.g., [63]), other studies reveal concerningly high percentages of cats in poor condition (e.g., [64,65]).

Zito et al. [48] used distance observation to assess the condition of a convenience sample of 676 cats (divided into n = 213 companion cats, n = 210 managed stray cats, and n = 253 unmanaged stray cats) from ‘various unspecified locations’ across Auckland. They identified the health conditions for managed and unmanaged stray cats: eye and nasal discharges, and injuries (‘trauma’ in the present study). Crusting around the ears was reported in 11.4% of stray cats [48] but was not one of the major categories of health problems in LM cats (present study). In contrast, snuffles, rattly chests, ringworm infections, and dental problems were commonly reported in the LM cats, as well as some cats that were FIV- or FeLV-positive, but these conditions were not recorded by [48]. Clinical notes for the LM cats indicated the diversity and severity of less common conditions, for example: *“Covered in fleas, more fleas than cat. Smaller (cat) dehydrated and both anaemic”*. Records also sometimes included comments on the fate of cats that did not reach the clinic, for example: *“post-partem (sic) cat spey, left flank as mammary tissue engorged (kittens killed by a dog)”*. Zito et al. [66] also reported that 23 of 429 cats (5.4%) trapped in their TNR trial were euthanized for one or more conditions, including ataxia, diarrhoea, oral ulcers, ringworm, being underage, upper respiratory tract infections, trauma, cancer, corneal ulcers, dehydration, emaciation, or FIV. Although the prevalence of euthanasia in [66] was less than that reported for LM, overall, the greater range of conditions requiring intervention when cats were handled cautions against using visual assessments alone.

The consistent pattern over 20 years of *c*. 80% of cats being less than a year old, and only *c*. 2.0% estimated to be over 5 years old, indicates short life expectancies or possibly trap-shyness in older cats. The high removal of older cats is less likely to be a factor, given that our data indicate that kittens are more likely to be rehomed. This low survival rate agrees with estimates of 75–90% mortality by six months of age for free-ranging kittens from high-density populations [7,62,67,68,69]. Sex ratios were close to parity in young kittens, but by adulthood were biased to females, before reverting to parity or male-biased ratios in older cats. This is consistent with roaming and fighting in males leading to higher mortality at early ages (e.g., data on the male-biased prevalence of FIV in stray or shelter cats, [20,61,70,71]), while older females might be exhausted by constant breeding (*c.* 23% of female LM cats neutered at the Kohimarama Veterinary Clinic were either pregnant or showed signs of supporting kittens). The maintenance of kitten numbers—despite TAR—implies migration, ongoing abandonment of cats, small numbers of cats not removed, breeding successfully, or that the number of cats processed was small relative to the population.

By contrast, the age profiles of owned cats include larger proportions of cats over five years old (e.g., [72,73,74]). However, even despite the care of their owners, pet cats display higher risk behaviours when young and are more likely to suffer trauma (Auckland: [75], elsewhere: [17]). This supports our interpretation of high mortality of young stray cats. Alternative explanations are that calls for LM to help are more likely in response to sightings of kittens; however, this would not explain the age-related shifts in the sex ratio of trapped cats. An alternative is that older animals are wary and trap-shy and, hence, less likely to be caught; this hypothesis requires further data on trappability.

Based on the LM data, we argue that there is a strong case on welfare grounds to reduce Auckland’s stray cat population. This finding also supports caution regarding neutering and returning cats. While some studies show improved welfare of TNR cats based on reductions in fighting [63,76], improved longevity [62], lower rates of infectious disease [6], and healthier appearance [63], we share the concerns of some animal welfare groups, such as People for the Ethical Treatment of Animals (PETA), that returned cats may not receive long-term caregiver support and are still highly vulnerable to trauma [77,78].

### 4.3. Comparing TAR, TNR, and TE

Given the significant welfare issues for stray cats themselves and the nuisance, public health risks and depredation of wildlife they may cause, intervention is needed. Modelling studies indicate that, regardless of the choice of management strategy, the intensity of application is critical to achieving a strong population reduction [44,79]. In this context, key features of TE, TNR, and TAR are summarised in Table 2.

If speedy population reduction is desired, then TE and TAR have the greatest potential for rapid results because cats are removed immediately. While adoption is an important component of TNR, unadopted cats are still returned, so a significant population reduction may be delayed or unachievable. Whether this is acceptable will depend on the urgency of resolving problems, such as wildlife impacts or public health risks, which are not mitigated by neutering alone [25,80]. TNR is attractive to many because euthanasia is reduced [8,43], although the prevalence of desexing pregnant cats may be higher than many realise [81]. This is an ethical concern [82], alongside the welfare of returned cats irrespective of whether or not there is caregiver support [77,78,83]. Whether TAR reduces public or veterinary health risks or reduces predation by cats, depends on the husbandry of the adopted cats by their new owners. If the cats are allowed to roam unrestricted, benefits may be limited [24,29]. Finally, adoption is assumed to be for the rest of the cat’s life [84]. Post-adoption follow-up studies could address this question for adoptions under both TAR and TNR (e.g., [85,86]), including the possibility that cats are assessed incorrectly as suitable for adoption.

Comparative data on the costs of different approaches to stray cat management are limited and complicated by whether or not they include costs for: ongoing maintenance of TNR colonies over the lifespans of the returned cats, discounting or pro bono work by veterinarians, volunteer labour, community education to increase desexing of owned pets and reduce abandonment, and valuing the benefits of particular interventions against costs [37,38,87]. Valuations of benefits are contentious, especially when values are placed on the wildlife lost to predation or cat-borne diseases [88]. Nevertheless, potential benefits that might be assessed are saved costs to public health, veterinary health, agricultural production from the curtailment of diseases transmitted by stray cats [89], wildlife conservation [25,26,27], and the improved mental health of animal welfare professionals who can be traumatised by frequently euthanising healthy animals [90,91]. Overall, the costs reported in the literature at best provide comparisons for a local situation. Before generalising, agreement is necessary on the costs of subsidies and volunteer efforts (including ongoing maintenance where provisioning of TNR colonies is established), benefit valuations, and the projected life of a control program.

**Table 2:** Comparisons of the main features of trap and euthanize (TE), trap–neuter–return (TNR), and trap–assess–resolve (TAR) for managing populations of unowned cats.

Feature	TE	TNR	TAR
Speed of population reduction	Rapid extirpation in closed populations but needs reapplication if abandonment or migration replenishes cats [37,87].	Some local successes are claimed, especially where populations are closed and there is high adoption within the program (e.g., [92,93]); however, Gunther et al. [60] observed that achieving sterilization rates of at least 75% (required for population decline) is ‘almost impossible to reach and sustain on a meta-population scale.’ Gunther et al. [46] reported population reductions of approximately 7% per year following high intensity (>70% neutering) maintained across contiguous sites covering a 20 km^2^ urban area.	Should be rapid in a closed population because cats are removed. Needs reapplication when numbers are replenished by migration or abandonment, similar to the problems noted for TE and for TNR that are not applied across contiguous areas simultaneously [46].
Addresses problems caused by stray cats	Yes, if applied at a level that reduces populations. Euthanized cats cannot breed, experience poor welfare, threaten wildlife, cause nuisance, or spread disease.	From a welfare perspective, prevents the birth of kittens likely to die young [44], but high numbers of kittens may be aborted when neutering pregnant queens [81]. May reduce disease transmission if it includes vaccination [6]. Neutered cats are less likely to fight or cause nuisances [6,63]. They still hunt, though, so that problem is unaddressed [25,26,46,94]. Some cats are adopted, gaining lasting care.	Provides veterinary care including vaccination and, for adoptable cats, provides lasting care in homes. The husbandry of the adopted cats will determine the levels of nuisances they may cause, the risk of them spreading diseases, and any threat to wildlife. Seo et al. [83,95] recommended adoption as a replacement for TNR for animal welfare and public health reasons.
Opportunities for citizens to be involved	None, assuming that citizens are unlikely to volunteer for trapping followed by euthanasia.	High, including trapping and transporting cats and providing food and shelter to colonies.	High, including trapping and transporting cats and fostering.
Euthanasia	All animals (other than pets returned to owners) are euthanized.	Greatly reduced. Only ill cats are euthanized.	Greatly reduced. Only ill or unsocialized cats are euthanized. However, there is a risk that timid cats are euthanized unnecessarily or that unsuitable cats are adopted.
Other ethical considerations	Fits within a utilitarian or consequentialist ethical approach, in which managers strive to achieve the best outcomes overall for all animals involved [96,97], which in this case would include the cats themselves, other organisms or people threatened with diseases, and wildlife at risk of predation.	Follows a deontological or rights approach respecting intrinsic animal rights, although ethically TNR must also justify neutering [98] and death of kittens when neutering pregnant queens [82]. TNR may also encourage dumping cats at TNR colonies [99,100]. Even when fed, cats returned to the site may have compromised welfare [7], see also this paper. TNR also values cats as a species over the wildlife they may hunt or infect with disease [101].	Similar to TNR in following a deontological approach, as well as needing to justify neutering (including of pregnant animals) from an ethical perspective [82,98]. Adopting cats, rather than returning them to the site, should provide a high level of care. Euthanasia of unadoptable cats can be argued to be preferable to the risks associated with returning them [102]. Whether adopted cats still threaten wildlife or spread disease is dependent on their husbandry.
Moral distress to veterinary and animal welfare professionals	Moral distress caused by euthanasia or leaving stray cats on the streets are only addressed if populations are suppressed long-term.	Euthanasia is greatly reduced but distress over unowned cats remains. Some moral stress may arise from neutering pregnant queens [82].	Euthanasia is greatly reduced (but not as much as TNR), while any distress over returning cats to the streets is eliminated. Some moral stress may arise from neutering pregnant queens [82].
Costs per cat	USD 52–123 [38]. USD 215.82 † [37]. ~Half the cost of TNR according to comparative modelling [87].	USD 20–97 [38]. ~Twice the cost of TE according to comparative modelling [87]. At least USD 45 [46], based on a cost of over USD 1 million for a program sustained over 9 years in a 20 km^2^ urban area that neutered 22,144 cats.	USD 104–550 [38]. USD 103.98 ‡ (this paper).

† AUD 277.5 at exchange rate (16 May 2021); ‡ NZD 142.50 at exchange rate (26 May 2021).

TAR is not without potential problems. Timid cats may be euthanized unnecessarily instead of proposed for adoption, or some cats may be recommended for adoption when they are temperamentally unsuited to be pets. The extent to which TAR ameliorates public health risks or hunting behaviour also depends on the husbandry of adopted cats. If they are kept on the owners’ properties, public health is enhanced, and hunting is curtailed. If adopted cats are allowed to roam freely, they may still pose health risks, hunt wildlife, and risk physical trauma themselves.

### 4.4. Integrated Responses to Reduce Populations of Stray Cats

Despite removing over 14,000 stray cats from the streets of Auckland, there is no sign of a decline in the need for LM services. We have no data showing reduced numbers of cats at managed locations, reduced numbers of locations with cats, or declining captures per unit effort. Thus, programs such as TAR should be implemented as part of a wider strategy, ideally conducted at the level of the municipality and involving all stakeholders, addressing the management of owned animals to prevent unwanted breeding, abandonment, and loss while simultaneously reducing the number of strays. One example of such an integrated approach is the Australian Capital Territory’s 2021–2031 Cat Plan [103], which seeks to achieve the vision that by 2031 ‘All cats in the ACT will be owned, wanted and cared for by responsible owners.’ Action 8 under that plan requires: ‘Work with animal care and rescue organisations to manage semi-owned and unowned cats in public places, through trap, de-sex and adopt activities’—TAR, as described in this paper.

The primary need is to reduce unwanted breeding. Neutering of owned animals, including increased use of early-age or prepubertal neutering (before six months) and mandatory neutering prior to the sale or transfer of registered animals, will prevent unplanned breeding and possible abandonment of unwanted animals [104,105]. Many of the concerns regarding the effects of neutering on the health and development of cats were rebutted with empirical data in the publications arising from Belgium’s Sterycat Program [106,107,108,109]; however, there remains evidence that even in countries reporting a high prevalence of neutering, many cats are not neutered until later in life [110,111,112]. Groups such as Australia’s National Neutering Network (https://ndn.org.au) or the United Kingdom’s Kitten Neutering Database (KiND, http://www.kind.cats.org.uk (31 August 2022)) may help to reverse this trend. Sumner et al. [43] report that the New Zealand Veterinary Association endorses prepubertal desexing.

Mandatory IDs, including both microchipping and collar-worn IDs, will assist by improving the low rates of returning lost cats to their owners [113,114]. Measures can also be taken to encourage people to own and keep pets, with signs that the adoption of a shelter cat is becoming desirable, at least in some countries [115]. There was even a surge in pet adoptions in relation to the COVID-19 pandemic [116]. More pet-friendly rental arrangements, better provision of information and services to owners (for example, regarding the welfare benefits of containment of cats on their owners’ property), as well as assistance to shelters in rehoming stray animals, may also assist [55,57,84]. If people are confident that cats surrendered to shelters are highly likely to be rehomed, they may not feed strays and thereby encourage unwanted breeding. Thus, rather than seek positive attitudes towards unowned cats alone [117], we would prefer that citizens have positive attitudes towards finding homes for unowned cats. We are divided as to whether it is ever good practice to feed strays. Some of us disagree with feeding because it may encourage the abandonment of animals at feeding stations, facilitate recruitment, and encourage rodents and other pests that take scraps. Others argue that feeding alleviates hunger and, although it makes trapping more difficult, it is better than cats suffering from starvation. LM neither encourages nor discourages people to feed strays.

In our opinion, TAR fits well within such an integrated strategy. In comparison to TE, it reduces euthanasia and addresses the concern expressed by some citizens regarding lethal control, although we acknowledge that we have no evidence for population reduction. Nevertheless, in comparison to TNR, it rehomes stray cats, potentially reducing a wide range of welfare problems and, with appropriate husbandry, concerns over nuisances, public health, and attacks on wildlife. Ultimately, the choice of approach in any situation rests with the local community [83,100].

## 5. Conclusions

The poor animal welfare outcomes, public health risks, and threats to wildlife often associated with unowned, free-roaming cats, demand action [8,36]. While this can be presented as a choice between euthanasia and TNR, the work of LM shows that TAR can also be considered. TAR reduces euthanasia considerably relative to culling, and adoption should raise the level of care well above life on the street. TAR’s effectiveness would be enhanced by education on the value of pre-pubertal desexing [118] and the problems caused by abandoning cats [83,100]. Further data are needed on its success in reducing populations and curtailing risks to wildlife and public health.

## Figures and Tables

**Table 1:** Cat colonies (total n = 3737) resolved by Lonely Miaow, Auckland, New Zealand, between June 1995 and June 2020.

Year Range:	July 1995–December 2010		January 2011–June 2020	
Location of Colony	Number (Percent) of Colonies		Number (Percent) of Colonies	
Residential property	1300	(79.1%)	1700	(81.2%)
Commercial premises	164	(10.0%)	154	(7.4%)
Peri-urban	69	(4.2%)	44	(2.1%)
Industrial site	29	(1.8%)	52	(2.5%)
Council subsidized housing	10	(0.6%)	57	(2.7%)
Hospital	15	(0.9%)	5	(0.2%)
Other (e.g., schools, beach, restaurants)	56	(3.4%)	82	(3.9%)
Sub-total	1643		2094	

## Data Availability

Most relevant data are reproduced in the figures and tables within the paper. The original Lonely Miaow (Inc.) data are confidential, as are the original case records we were permitted to examine for the study.

## Supplementary Materials

The following supporting information can be downloaded at: https://www.mdpi.com/article/10.3390/ani12172301/s1, Table S1: Examples of forms used over the years to assess the health and temperament of trapped cats.
