# Supplementary material for: Intensive Adoption as a Management Strategy for Unowned, Urban Cats: A Case Study of 25 Years of Trap–Assess–Resolve (TAR) in Auckland, New Zealand
Source: Animals (Basel). 2022 Sep 5;12(17):2301. doi: 10.3390/ani12172301 (PMC9454951; doi:10.3390/ani12172301)
Supplement: Supplementary file 1 [file animals-12-02301-s001.zip › animals-1881052-supplementary.pdf]

Table S1 Examples of reporting forms used by Lonely Miaow over the years to assess the health and temperament of trapped cats. An interviews on procedures used in trapping and assessment is available at: https://www.radionz.co.nz/search/results?utf8=%E2%9C%93&q=Spectrum+6+June+1999&commit=Search.

THE LONELY MIAOW

ASSOCIATION INC.

VETERINARY ADMITTANCE FORM

ID number: ______________________ Contact person: ______________________

Name: ______________________ Contact phone: ______________________

Colour: ______________________ Authorisation: ______________________

Sex: ______________________ Age: ______________________

Suburb trapped: ______________________ Date trapped: ______________________

Admission date: ______________________ Collection date: ______________________

Temperament assessment: Wild Rehomeable

Health check:

☐ Ringworm

☐ Eye infection

☐ Other ______________________

Health action:

☐ Worm ☐ Desex

☐ Deflea ☐ Vaccinate

☐ Euthanasia

Medical problems and treatment instructions: ______________________

____

____

Date discharged: ______________________ Veterinarian signature: ______________________

**** PLEASE ENTER ID NUMBER AND SEX ONTO INVOICE STATEMENT.
